# Prognostic value of depression and anxiety on colorectal cancer-related mortality: a systematic review and meta-analysis based on univariate and multivariate data

**DOI:** 10.1007/s00384-024-04619-6

**Published:** 2024-04-02

**Authors:** Shijun Xia, Yuwen Zhu, Lidan Luo, Wenjiang Wu, Lijuan Ma, Linchong Yu, Yue Li

**Affiliations:** 1 Shenzhen Hospital of Guangzhou University of Chinese Medicine＜Futian＞, Shenzhen, 518000 Guangdong China; 2 Shenzhen Traditional Chinese Medicine Anorectal Hospital＜Futian＞, Shenzhen, 518000 Guangdong China

**Keywords:** Depression, Anxiety, Colorectal cancer, Mortality, Meta-analysis

## Abstract

**Background:**

Depression and anxiety are common mental disorders in patients with colorectal cancer (CRC); however, it remains unclear whether they are related to cancer mortality.

**Method:**

Based on a systematic literature search, 12 eligible studies involving 26,907 patients with CRC were included in this study.

**Results:**

Univariate analysis revealed that anxiety was associated with an all-cause mortality rate of 1.42 (1.02, 1.96), whereas multivariate analysis revealed that anxiety was not associated with an all-cause mortality rate of 0.73 (0.39, 1.36). In univariate and multivariate analyses, depression was associated with all-cause mortality rates of 1.89 (1.68, 2.13) and 1.62 (1.27, 2.06), respectively, but not with the cancer-associated mortality rate of 1.16 (0.91, 1.48) in multivariate analyses. Multivariate subgroup analysis of depression and all-cause mortality showed that younger age (≤65 years), being diagnosed with depression/anxiety after a confirmed cancer diagnosis, and shorter follow-up time (<5 years) were associated with poor prognosis.

**Conclusions:**

Our study emphasizes the key roles of depression and anxiety as independent factors for predicting the survival of patients with CRC. However, owing to the significant heterogeneity among the included studies, the results should be interpreted with caution. Early detection and effective treatment of depression and anxiety in patients with CRC have public health and clinical significance.

## Introduction

Colorectal cancer (CRC) is the third most common cancer worldwide and the second leading cause of cancer death [1]. Although the number of long-term survivors of CRC has increased due to advances in testing and treatment, approximately 50% of the population has reported high mortality rates within 10 years of diagnosis [2]. Multiple factors are known to affect the survival rate of patients with CRC. Certain immutable factors are associated with reduced survival rate, such as cancer characteristics, including advanced and proximal tumors [3]. However, > 50% of cases and deaths can be attributed to modifiable risk factors, such as smoking, unhealthy diet, excessive alcohol consumption, lack of exercise, and being overweight, making prevention possible [4]. Thus, it is crucial to identify the modifiable factors that affect CRC-related mortality to improve prognosis and overall survival in patients.

Mental disorders are one of the main causes of disability and premature death worldwide [5]. Depression and anxiety are the most common mental disorders, with prevalence rates of 5% and 7% in the general population over the past year [6]. Importantly, depression and anxiety are more common among patients with cancer, affecting 20% and 10% of them, respectively [7]. The impact of CRC on the body and its functions may have further adverse psychological effects on the overall health and quality of life of patients. Therefore, patients with CRC suffer from negative emotions under chronic psychological pressure. In 2019, Peng et al. [8] published a literature review of 15 studies and reported that the prevalence rates of anxiety and depression in patients diagnosed with CRC were 1.0–47.2% and 1.6–57%, respectively. Studies have also shown that patients with CRC have a 51% increased risk of depression after diagnosis (pooled hazard ratio (HR), 1.51; 95% confidence interval (CI), 1.10–2.09) but have no association with anxiety (pooled HR, 1.43; 95% CI, 0.79–2.57) [9]. In addition, in 2021, a cohort study in Denmark reported that patients with CRC had a significantly higher risk of depression than cancer-free individuals, even after participating in the study for 5 years (HR, 2.65; 95% CI, 1.61–4.36) [10]. Although psychological factors are widely known to predict colorectal cancer-associated mortality, studies have reported inconsistent results; some studies have revealed that depression is associated with an increase in all-cause mortality [2, 11, 12], whereas others have indicated that it is not associated with mortality [13]. Similarly, studies have shown inconsistent relationships between anxiety and all-cause mortality in patients with CRC, with some studies suggesting a correlation [14, 15], whereas another study showing no correlation [16]. Moreover, conflicting conclusions have been drawn regarding the specific mortality rate of patients with CRC [13, 17]. Thus, there is a need for comprehensive and rigorous research to determine the relationship of depression and anxiety with the survival and progression of patients with CRC.

Therefore, we conducted a comprehensive and rigorous meta-analysis to understand the relationship of depression and anxiety with CRC.

## Methods

### Protocol and guidance

This study was conducted based on the PRISMA (Preferred Reporting Items for Systematic Reviews and Meta-Analyses) [18] and AMSTAR (Assessing the methodological quality of systematic reviews) [19] guidelines. The protocol for this review has been registered with PROSPERO (CRD42023414631). The need for ethical approval or informed consent in this study was waived.

### Search strategy

Based on the recommendations of the Meta-analysis of Observational Studies in Epidemiology group [20], we searched the following electronic databases for articles written in English published from inception until March 26, 2023: PubMed, Embase, and the Cochrane Library. The following search terms were used: (“colon cancer” or “colon tumor” or “colorectal cancer” or “colorectal cancer”) and (“depression” or “anxiety”) and (“survival” or “mortality” or “metastasis” or “recurrence”). The search strategy was implemented using a combination of index words and free text keywords. In addition, the reference lists in these articles were evaluated to include more comprehensive studies.

### Inclusion and exclusion criteria

The criteria for the inclusion of studies were as follows: (1) cohort study, (2) survey of patients with CRC, (3) assessment of depression or anxiety according to standard diagnostic criteria or self-reported scale, and (4) provision of HRs or risk ratios and 95% CIs for all-cause or cancer-specific mortality. Further, the exclusion criteria were as follows: (1) studies involving participants with malignant tumors other than CRC, (2) studies involving patients with cancer but without depression or anxiety, and (3) studies with insufficient data.

The data were independently extracted by two authors (SJX and LJM). In the event of disputes and disagreements, a third author (LCY) was consulted to reach a consensus. This review collected the following details: name of the lead author, year of publication, country, number of participants, age, time of depression assessment, depression/anxiety measurement method, and follow-up time.

All selected articles were examined using the quality assessment scale of the Newcastle Ottawa cohort study. This semiquantitative scale uses a star rating system to evaluate the quality of eight projects in three areas: selection (four projects, one star per project), comparability (one project, up to two stars), and exposure (three projects, one star per project). In this meta-analysis, we classified the quality as good (≥ 7 stars), average (4–6 stars), or poor (< 4 stars).

The outcomes were all-cause (death from any cause) and CRC-specific (death due to CRC as well as depression and anxiety) mortality rates of patients with CRC. This assessment is based on the HRs and 95% CIs obtained from each study. Most HRs are adjusted for different variables, such as age or tumor staging. Unadjusted HR for univariate analysis; conduct a multivariate analysis on the adjusted HR. Subgroup analysis was conducted based on age (mean age, ≤ 65 vs. > 65 years), follow-up time (< 5 vs. ≥ 5 years), and depression assessment time (before vs. after cancer diagnosis).

Review Manager version 5.3 (North Cochrane Center, Cochrane Collaboration, London, UK) was used for data analysis. HR was used as a measure of effectiveness at 95% CI. Based on *I*^2^ values, four categories of heterogeneity were established: no heterogeneity (*I*^2^ < 25%), low heterogeneity (25% ≤ *I*^2^ < 50%), moderate heterogeneity (50% ≤ *I*^2^ < 75%), and high heterogeneity (*I*^2^ ≥ 75%).

## Results

### Eligible studies and study characteristics

**Fig. 1:**
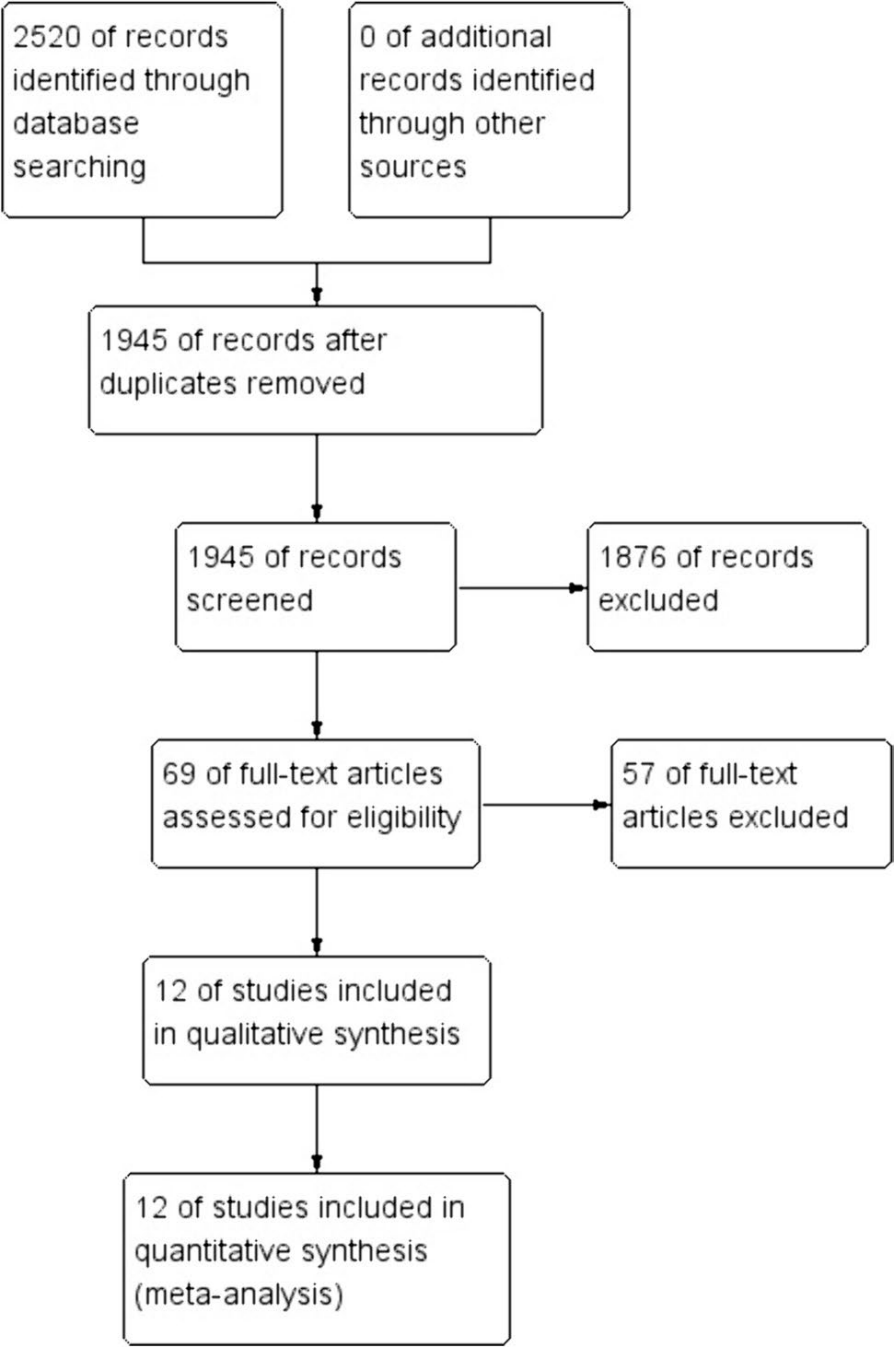
Flow chart of identification of eligible studies

After identifying 2520 references, 575 duplicate publications and 1 876 unrelated studies were excluded, leaving 69 studies that potentially met the inclusion and exclusion criteria. Finally, 12 cohort studies conducted between 2013 and 2023 were included in this meta-analysis [2, 11–17, 21–24] (Fig. 1).

Table 1 lists the general characteristics of the 12 included studies. These studies included 26,907 patients with CRC, with 302–8961 patients per study. Six prospective studies and 6 retrospective studies. The shortest follow-up time was 1.9 years, whereas the longest follow-up time exceeded 20 years. Among these studies, five studies were from Europe, four were from the USA, two were from Asia, and one was from Australia. Mental symptoms were evaluated in these studies using various tools, such as the Hospital Anxiety and Depression Scale, Epidemiological Research Center for Epileptic Studies Depression Scale, International Classification of Diseases 9, Crown Crisp Index, 7-item Generalized Anxiety Disorder, and Geriatric Depression Scale Short Form. In most studies, adjustment was performed for variables that affect the risk of cancer death, such as age, tumor stage, and comorbidities. Multivariate analysis was conducted for the adjusted data, whereas univariate analysis was conducted for the unadjusted data.

**Table 1:** Characteristics of included trials

**First author (publication year)**	**Country**	**Design type**	**Number of participants**	**Age (years) (means)**	**Mental stress before/after cancer diagnosis**	**Tool**	**Follow-up duration (years)**	**Quality assessment**
Mols (2013) [12]	Netherlands	Prospective	1074	67	After	HADS	10 years	9
Rane (2014) [17]	USA	Retrospective	2119	-	Before	-	3 years	-
Schofield (2016) [21]	Australia	Prospective	429	67	Before	HADS	2.5 years	8
Lloyd (2019) [11]	USA	Retrospective	8961	-	After	ICD-9	> 5 years	8
Trudel-Fitzgerald (2020) [2]	USA	Prospective	1732	> 70	After	CES-D、CCI、GAD-7、GDS-SF	> 20 years	9
Liang (2020) [13]	USA	Prospective	2396	-	Before	CES-D	8.8 years	9
Walker (2020) [16]	UK	Retrospective	1573	65	After	HADS	1.9 years	8
Xia (2020) [15]	China	Retrospective	381	59.4	After	HADS	3 years	7
Walker (2021) [22]	UK	Retrospective	2807	65	Before	HADS	< 3 years	7
Zhou (2021) [23]	China	Retrospective	302	63.7	After	HADS	3 years	8
Varela-Moreno (2022) [24]	Spain	Prospective	2602	68	After	HADS	5 years	9
Orive (2023) [14]	Spain	Prospective	2531	-	After	HADS	5 years	6

*HADS* Hospital Anxiety and Depression Scale, *ICD-9* International Classification of Diseases 9, *CES-D* Epidemiological Research Center for Epileptic Studies Depression Scale, *CCI* Crown Crisp Index, *GAD-7* 7-item Generalized Anxiety Disorder, *GDS-SF* Geriatric Depression Scale Short Form

According to the quality evaluation criteria, ten studies were rated to be of good quality and one study was rated to be of average quality; further, one study could not be evaluated (Table 2).

**Table 2:** Quality assessment of the included cohort studies using the NOS scale

**First author**	**Selection**				**Comparability**^e^	**Outcome**			**Total**
	**Representativeness**^**a**^	**Selection**^**b**^	**Ascertainment**^**c**^	**Demonstration**^**d**^		**Assessment**^**f**^	**Follow-up**^**g**^	**Adequacy**^**h**^	
Mols [12]	☆	☆	☆	☆	☆☆	☆	☆	☆	9
Rane [17]	-	-	-	-	-	-	-	-	-
Schofield [21]	☆	☆	☆	-	☆☆	☆	☆	☆	8
Lloyd [11]	☆	☆	☆	-	☆☆	☆	☆	☆	8
Trudel-Fitzgerald [2]	☆	☆	☆	☆	☆☆	☆	☆	☆	9
Liang [13]	☆	☆	☆	☆	☆☆	☆	☆	☆	9
Walker [16]	☆	☆	☆	-	☆☆	☆	☆	☆	8
Xia [15]	☆	☆	☆	-	☆	☆	☆	☆	7
Walker [22]	☆	☆	☆	-	☆☆	☆	-	☆	7
Zhou [23]	☆	☆	☆	-	☆☆	☆	☆	☆	8
Varela-Moreno [24]	☆	☆	☆	☆	☆☆	☆	☆	☆	9
Orive [14]	☆	☆	☆	-	☆	☆	☆	-	6

^a^Representativeness of the exposed cohort

^b^Selection of the non-exposed cohort

^c^Ascertainment of exposure

^d^Demonstration that outcome of interest was not present at start of study

^e^Comparability of cohorts on the basis of the design or analysis

^f^Assessment of outcome

^g^Was follow-up long enough for outcomes to occur

^h^Adequacy of follow-up of cohorts

### Impact of anxiety on mortality in patients with CRC

#### All-cause mortality rate

**Fig. 2:**
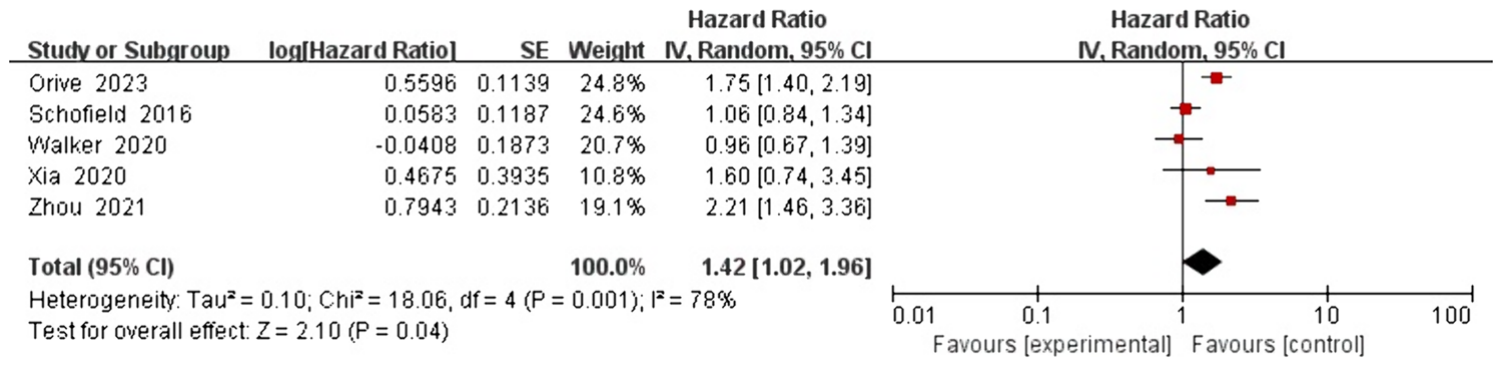
Impact of anxiety on all-cause mortality rate based on univariate analysis

**Fig. 3:**
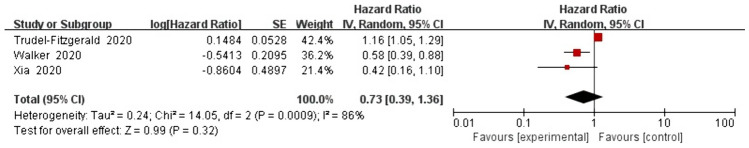
Impact of anxiety on all-cause mortality rate based on multivariate analysis

For the association between anxiety and all-cause mortality, the five studies analyzed in the univariate analysis based on the random effects model yielded a pooled HR of 1.42 (1.02, 1.96), with high heterogeneity (*I*^2^ = 78% and *P* = 0.001; Fig. 2). For the same association, the three studies analyzed in the multivariate analysis based on the random effects model yielded a pooled HR of 0.73 (0.39, 1.36), with high heterogeneity (*I*^2^ = 86% and *P* = 0.0009; Fig. 3).

Univariate analysis revealed that anxiety was associated with a 42% increased risk of all-cause mortality. In contrast, multivariate analysis indicated that anxiety was not associated with the risk of all-cause mortality.

### Impact of depression on mortality in patients with CRC

#### All-cause mortality rate

**Fig. 4:**
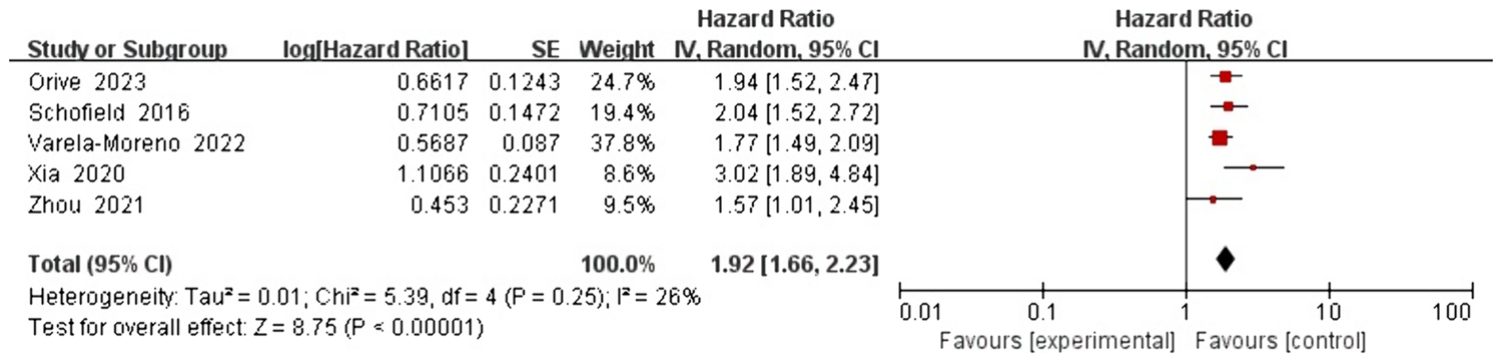
Impact of depression on all-cause mortality rate based on univariate analysis

**Fig. 5:**
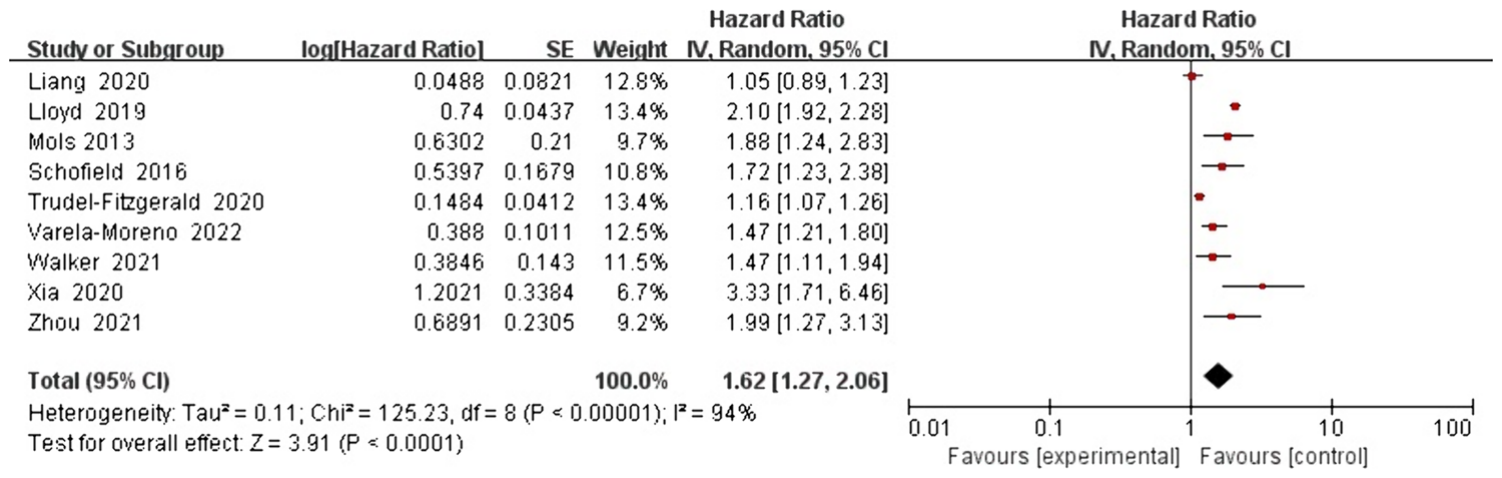
Impact of depression on all-cause mortality rate based on multivariate analysis

For the association between depression and all-cause mortality, the five studies analyzed in the univariate analysis based on the fixed effects model yielded a pooled HR of 1.89 (1.68, 2.13), with low heterogeneity (*I*^2^ = 26% and *P* = 0.25; Fig. 4). For the same association, the nine studies analyzed in the multivariate analysis based on the random effects model yielded a pooled HR of 1.62 (1.27, 2.06), with high heterogeneity (*I*^2^ = 94% and *P* < 0.00001; Fig. 5). Univariate analysis revealed that depression was associated with an 89% increased risk of all-cause mortality. However, multivariate analysis indicated that depression was associated with a 62% increased risk of all-cause mortality.

**Fig. 6:**
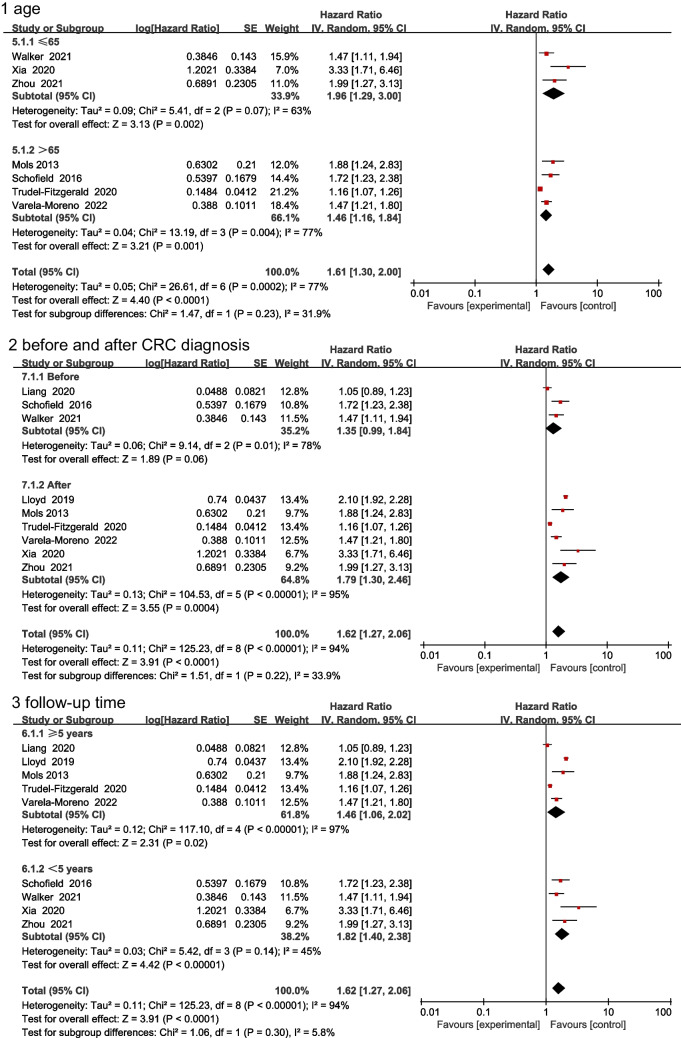
Impact of depression on all-cause mortality rate based on multivariate subgroup analysis. Age (**1**), before and after CRC diagnosis (**2**), and follow-up time (**3**)

In the multivariate analysis, the mean age of patients in three studies was ≤ 65 years, whereas that in four other studies was > 65 years. Three studies evaluated depression before CRC diagnosis, whereas six studies evaluated it after CRC diagnosis. The follow-up time in five studies was ≥ 5 years, whereas it was < 5 years in four other studies. Subgroup analysis was conducted based on age, time of depression assessment, and follow-up time. The results showed a significant correlation between patients aged ≤ 65 and > 65 years (1.96 (1.29, 3.00) vs. 1.46 (1.16, 1.84)) (Fig. 6(1)). A significant different correlation was observed in patients before and after CRC diagnosis (1.35 (0.99, 1.84) vs. 1.79 (1.30, 2.46)) (Fig. 6(2)). However, no significant difference in risk was noted between studies with a follow-up time of ≥ 5 years (1.46 (1.06, 2.02)) and those with a follow-up time of < 5 years (1.82 (1.40, 2.38)) (Fig. 6(3)).

### CRC-specific mortality rate

**Fig. 7:**
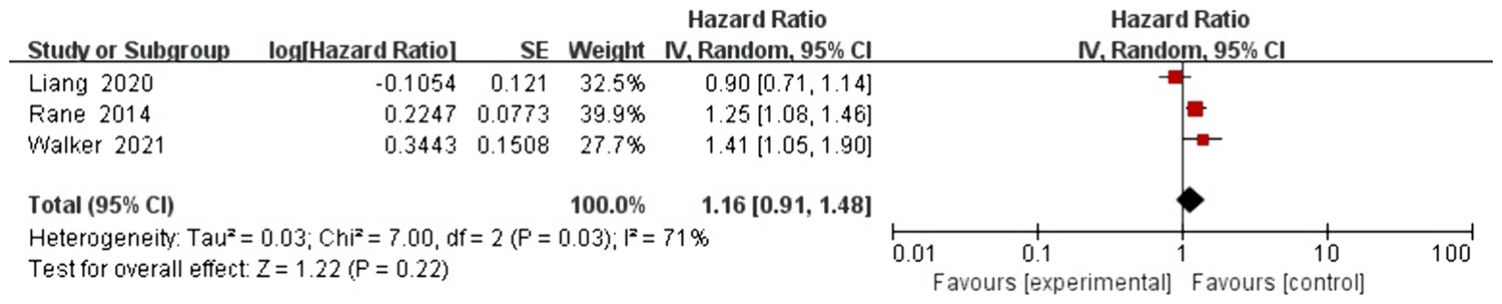
Impact of depression on CRC-specific mortality rate based on multivariate analysis

Three studies were included in the multivariate analysis. Based on the random effects model, the pooled HR for the association between depression and CRC-specific mortality was 1.16 (0.91, 1.48), with moderate heterogeneity (*I*^2^ = 71% and *P* = 0.03; Fig. 7). Multivariate analysis showed that depression was not associated with the risk of CRC-specific mortality.

### Publication bias and sensitivity analysis

In accordance with the criteria of the Cochrane Handbook for systematic reviews of interventions, publication bias was not analyzed because none of the groups comprised > 10 studies. However, sensitivity analysis was performed to evaluate the stability of the results, resulting in the omission of one study from the meta-analysis at a time. The results revealed no significant change in the corresponding merged estimates, indicating that no single study had an impact on the following results: multivariate analysis for the association between anxiety and all-cause mortality, univariate analysis for the association between depression and all-cause mortality, multivariate analysis for the association between depression and all-cause mortality, and multivariate analysis for the association between depression and CRC-specific mortality. However, a significant difference was noted in the results of univariate analysis for the association between anxiety and all-cause mortality. The results of sensitivity analysis are presented in Table 3.

**Table 3:** Sensitivity analysis results after removing one study at a time

Removed study	HR	95% CI	*P*_heterogeneity_	*I*^2^
a. Univariate analysis for the association between anxiety and all-cause mortality				
Orive et al. [14]	1.32	0.90–1.94	0.01	73%
Schofield et al. [21]	1.55	1.08–2.24	0.02	71%
Walker et al. [16]	1.57	1.09–2.25	0.003	78%
Xia et al. [15]	1.40	0.97–2.00	0.0005	83%
Zhou and Sun [23]	1.27	0.91–1.78	0.005	76%
b. Multivariate analysis for the association between anxiety and all-cause mortality				
Trudel-Fitzgerald et al. [2]	0.55	0.38–0.81	0.55	0%
Walker et al. [16]	0.79	0.30–2.06	0.04	76%
Xia et al. [15]	0.85	0.43–1.66	0.001	90%
c. Univariate analysis for the association between depression and all-cause mortality				
Orive et al. [14]	1.88	1.65–2.15	0.15	44%
Schofield et al. [21]	1.87	1.64–2.12	0.16	41%
Varela-Moreno et al. [24]	2.02	1.72–2.37	0.25	28%
Xia et al. [15]	1.84	1.63–2.07	0.72	0%
Zhou and Sun [23]	1.92	1.70–2.17	0.20	36%
d. Multivariate analysis for the association between depression and all-cause mortality				
Liang et al. [13]	1.73	1.33–2.24	< 0.00001	93%
Lloyd et al. [11]	1.49	1.24–1.78	< 0.0001	78%
Mols et al. [12]	1.59	1.23–2.06	< 0.00001	94%
Schofield et al. [21]	1.61	1.24–2.09	< 0.00001	94%
Trudel-Fitzgerald et al. [2]	1.70	1.322.18	< 0.00001	89%
Varela-Moreno et al. [24]	1.65	1.25–2.16	< 0.00001	94%
Walker et al. [16]	1.64	1.26–2.14	< 0.00001	94%
Xia et al. [15]	1.54	1.20–1.97	< 0.00001	94%
Zhou and Sun [23]	1.59	1.23–2.05	< 0.00001	94%
e. Multivariate analysis for the association between depression and colorectal cancer specific mortality				
Liang et al. [13]	1.28	1.12–1.47	0.48	0%
Rane et al. [17]	1.12	0.72–1.73	0.02	82%
Walker et al. [22]	1.08	0.78–1.48	0.02	81%

## Discussion

Previous studies have confirmed that anxiety and depression are related to cancer mortality; however, to the best of our knowledge, no study has analyzed their relationship with all-cause mortality in patients with CRC [25]. Thus, we performed a meta-analysis to investigate the association of anxiety and depression with CRC along with their predictive factors, but we did not compile or analyze data on mortality [26]. Our meta-analysis is the first study to investigate the predictive value of depression and anxiety on CRC-related mortality. Based on univariate and multivariate data, we conducted a meta-analysis of 26,907 patients and empirically demonstrated that depression and anxiety are associated with an increased risk of mortality in patients with CRC. In these patients, univariate analysis revealed that anxiety predicted a 42% increased risk of mortality, and depression predicted an 89% increased risk of mortality. Multivariate analysis revealed that depression predicted a 62% increased risk of mortality, but anxiety was not associated with an increased risk of mortality. Further, depression was not associated with an increased risk of CRC-specific mortality. Our study results indicate that depression is a more severe risk factor for CRC-specific mortality.

Depression and anxiety can affect the physiological function, treatment compliance, psychological function, and quality of life of patients with CRC, but the extent to the outcome of CRC remains unclear. Researchers have proposed several possible mechanisms, such as lifestyle, behavioral factors, and biological factors, to explain the association of increased mortality with depression and anxiety in patients with CRC. First, individuals with depression and anxiety are more likely to have unhealthy lifestyles, including prolonged sitting, increased alcohol consumption and smoking, poor diet, and obesity [27]. Second, treatment noncompliance is a serious behavioral factor, e.g., low adherence of patients with cancer to medical appointments and treatment [28], which may lead to a poor prognosis. Third, regarding biological factors, a previous showed that depression and anxiety may directly affect endocrine and immune processes [29]. The imbalance of the hypothalamic pituitary adrenal axis may mediate the susceptibility to hormone-related cancer. The activity of natural killer cells and DNA repair enzymes, which play important roles in cancer defense, is inhibited in patients with depression [30]. Further, the distinct effects of depression and anxiety on CRC may be due to their unique mechanisms. Depression manifests as depressive emotions, slow thinking, and loss of interest. In contrast, the characteristics of anxiety are prominent nervousness, worry, and a sense of anxiety. Patients with depression are also more likely to commit suicide than those with anxiety, which may lead to higher all-cause mortality rates.

Our study classified unadjusted and adjusted data as univariate and multivariate data. Considering that the mortality rate of patients with cancer is related to multiple factors, most reported studies have adjusted for demographic and tumor-related variables, such as age, sex, body mass index (BMI), tumor stage, and tumor size [11–13], whereas other studies have adjusted for anxiety and depression [16]. Regarding anxiety and risk of mortality in patients with CRC, univariate analysis suggested that anxiety is associated with an increased risk of mortality, whereas multivariate analysis suggested no correlation. Meanwhile, regarding depression and risk of mortality in patients with CRC, both univariate and multivariate analyses suggested that depression is associated with an increased risk of mortality. This indicates that depression is a stable predictor.

In multivariate analysis, we conducted subgroup analysis for the association between depression and mortality in terms of age, time of assessment, and follow-up time. The results showed a significant correlation between patients aged ≤ 65 years and those aged > 65 years. A significant different correlation was observed before and after CRC diagnosis. Further, no significant difference in risk was noted between studies with a follow-up time of ≥ 5 years and those with a follow-up time of < 5 years. Our study results showed that younger patients have a higher risk of death than older patients. This finding is consistent with that reported by Wang et al. [31], who analyzed the relationship between breast cancer and depression. However, based on the association between depression and various cancers, Pinquart et al. [32] indicated that the correlation between depression and mortality in older patients is stronger than that in younger patients. Considering that our research focused on CRC, the inconsistency in the results may indicate variation in the mechanisms of mortality due to different cancer types. Furthermore, the survival rate of patients with depression evaluated after cancer diagnosis was worse than that evaluated before cancer diagnosis. We considered that the early diagnosis of depression is associated with early treatment, which is key to improving survival rates. Compared with studies with a follow-up time of > 5 years, short-term follow-up studies reported higher mortality rates, which is consistent with the findings of Watson et al. [33]. This may be because the severity of depression decreases over time [34].

This meta-analysis has certain limitations. First, as mentioned above, the mortality rate of patients with cancer is influenced by various factors, such as age, BMI, tumor stage, and tumor size, which may affect the associations of anxiety and depression with cancer mortality. However, adjustments for different variables were performed in the included studies; therefore, it is not possible to achieve a consolidated association based on consistent adjustment for the same variables. Second, owing to the use of various tools to evaluate depression/anxiety in the included studies, we could not classify depression/anxiety based on different levels or conduct subgroup analysis.

Our research indicates that both depression and anxiety have adverse effects on all-cause mortality in patients with CRC and that depression cannot predict cancer-specific mortality. These findings confirm the importance of early detection and timely treatment of mental disorders in patients with CRC, especially in young patients and those in the early stages of cancer. Our study emphasizes the importance of depression and anxiety in predicting the prognosis of CRC and suggests that routine screening for mental disorders in patients with CRC should be carefully considered.

## Data Availability

All relevant data are within the paper. We searched the following electronic databases from PubMed, Embase, and the Cochrane Library.

## Declarations

### Provenance and peer review

Not commissioned, externally peer-reviewed.
